# Case Report: Non-ossifying fibromas with pathologic fractures in a patient with *NONO*-associated X-linked syndromic intellectual developmental disorder

**DOI:** 10.3389/fgene.2023.1167054

**Published:** 2023-07-18

**Authors:** Karin Writzl, Blaž Mavčič, Aleš Maver, Alenka Hodžić, Borut Peterlin

**Affiliations:** ^1^ Clinical Institute of Genomic Medicine, University Medical Centre Ljubljana, Ljubljana, Slovenia; ^2^ Faculty of Medicine, University of Ljubljana, Ljubljana, Slovenia; ^3^Department of Orthopaedic Surgery, University Medical Centre Ljubljana, Ljubljana, Slovenia

**Keywords:** *NONO*, non-ossifying fibromas, left ventricular non-compaction cardiomyopathy, X-linked intellectual disability syndrome, thrombocytopenia, recurrent fractures

## Abstract

The *NONO* gene encodes a nuclear protein involved in transcriptional regulation, RNA synthesis and DNA repair. Hemizygous loss-of function, *de novo* or maternally inherited variants in *NONO* have been associated with an X-linked syndromic intellectual developmental disorder-34 (OMIM # 300967), characterized by developmental delay, intellectual disability, hypotonia, macrocephaly, elongated face, structural abnormalities of corpus callosum and/or cerebellum, congenital heart defect and left ventricular non-compaction cardiomyopathy. Few patients have been described in the literature and the phenotype data are limited. We report a 17-year-old boy with dolihocephaly, elongated face, strabismus, speech and motor delay, intellectual disability, congenital heart defect (ASD, VSD and Ebstein’s anomaly), left ventricular non-compaction cardiomyopathy, bilateral inguinal hernia and cryptorchidism. Additional features included recurrent fractures due to multiple non-ossifying fibromas, thrombocytopenia, and renal anomalies. Exome sequencing revealed a *de novo* pathogenic variant (NM_001145408.2: c.348+2_ 348+15del) in intron 5 of the *NONO* gene. Renal anomalies and thrombocytopenia have been rarely reported in patients with *NONO*—X-linked intellectual disability syndrome, while recurrent fractures due to multiple non-ossifying fibromas have not previously been associated with this syndrome. The phenotypic spectrum of *NONO*—X-linked intellectual disability syndrome may be broader than currently known.

## 1 Introduction

The *NONO*-associated X-linked syndromic intellectual developmental disorder-34 (NONO-XLID) is a rare genetic disorder first described 8 years ago (Mircsof et al., 2015). It is caused by hemizygous loss-of-function variants in the non-POU domain containing, octamer-biding gene, *NONO*, which encodes an RNA and DNA binding protein involved in RNA synthesis, transcriptional regulation and DNA repair (Mircsof et al., 2015). The disorder is characterized by developmental delay, intellectual disability, macrocephaly and distinctive facial features, structural brain and heart anomalies and left ventricular non-compaction cardiomyopathy. Skeletal abnormalities were rarely described in patients and included kyphoscoliosis and planovalgus but not non-ossifying fibromas (Sewani et al., 2020).

Non-ossifying fibroma (NOF) is the most common bone tumor, thought to affect around 30%–40% of children and adolescents (Choi and Ro, 2021). It is a self-limiting benign tumor, usually diagnosed as an incidental finding on X-rays, that usually regresses after puberty (Choi and Ro, 2021).

Here we report a patient with a diagnosis of NONO-XLID, with characteristic clinical features confirmed by the presence of a germline pathogenic variant in *NONO*, who also suffered recurrent fractures due to multiple non-ossifying fibromas not previously reported in these patients, and thrombocytopenia and renal anomalies rarely reported patients with NONO-XLID. This has implications for clinical evaluation and management of patients with NONO-XLID.

## 2 Clinical report

The patient was a 17-year-old boy of Caucasian European origin, the second child of healthy non-consanguineous parents, who had an older healthy sister and a younger healthy brother. Family history is unremarkable. During pregnancy, hydronephrosis was noted. He was born at 38 weeks of gestation, after a normal vaginal delivery. Birth weight was 3.0 kg (−0.78 z), length 50 cm (−0.64 z), and head circumference 36 cm (0.98 z). Apgar 9/9. Shortly after birth, abdominal ultrasound revealed bilateral kidney dysplasia with bilateral pyeloureteral stenosis and vesicoureteral reflux. Echocardiogram revealed atrial septal defect (ASD), ventricular septal defect (VSD), Ebstein anomaly and spongoformic cardiomyopathy. Brain ultrasound revealed hypoplastic corpus callosum. He was noted to have thrombocytopenia.

His development was delayed; he started to walk at 4 years and spoke his first words at 6 years. He was toilet trained at 6.5 years. The patient’s history also included hearing loss, strabismus, and surgically-corrected bilateral inguinal hernia and cryptorchidism at the age of 2 years.

**FIGURE 1:**
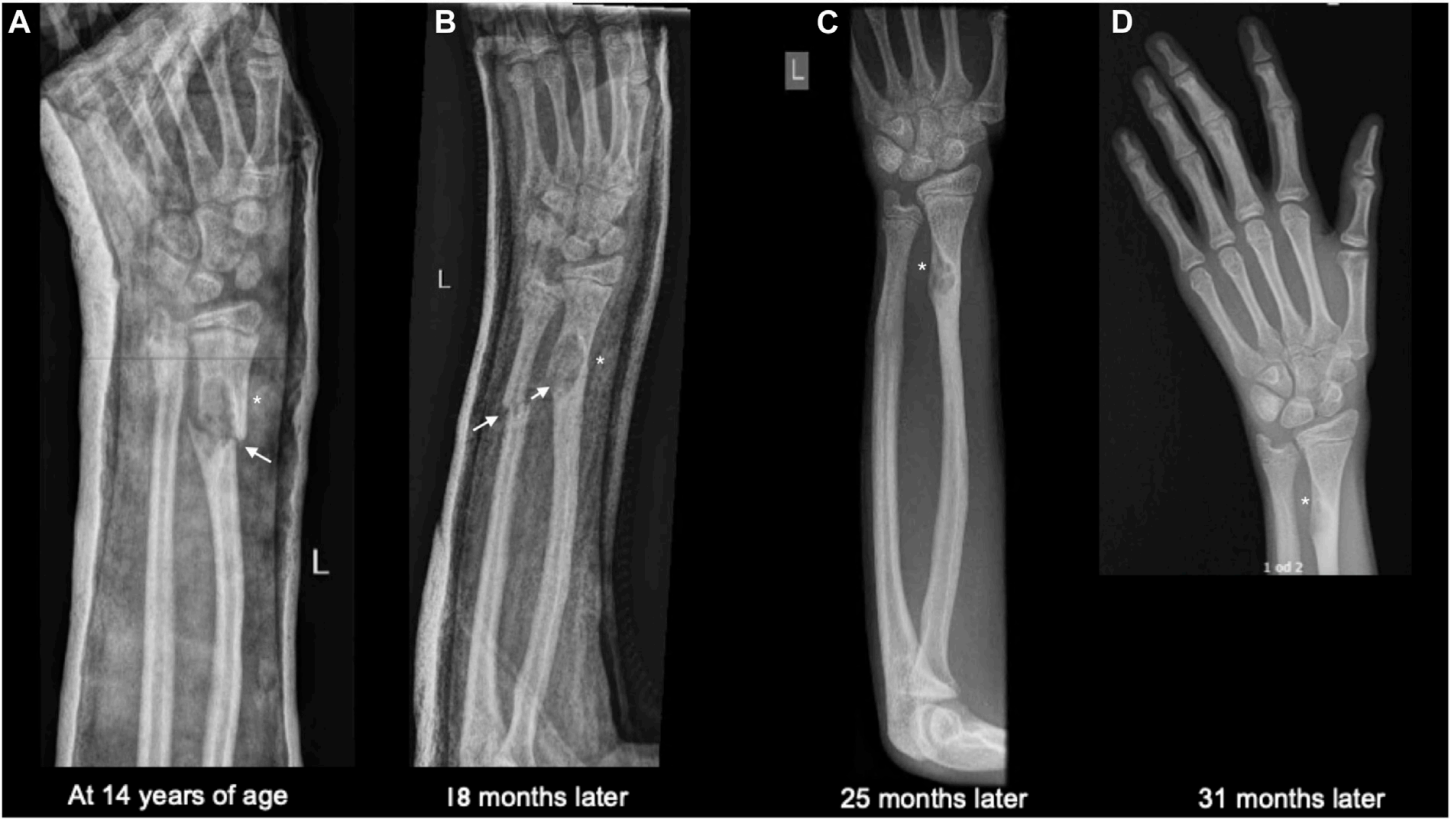
**(A)** Trauma anteroposterior radiograph of the right upper extremity showing a fracture through lesion in the distal radius and a fracture of ulna. **(B)** Trauma anteroposterior radiograph showing second fracture in the same area 18 months later. **(C,D)** Anteroposterior radiograph of the upper extremity 25 months **(C)** and 31 months **(D)** after the first injury showing united fracture. Fractures are indicated by arrows and non-ossifying fibromas by asterisks.

**FIGURE 2:**
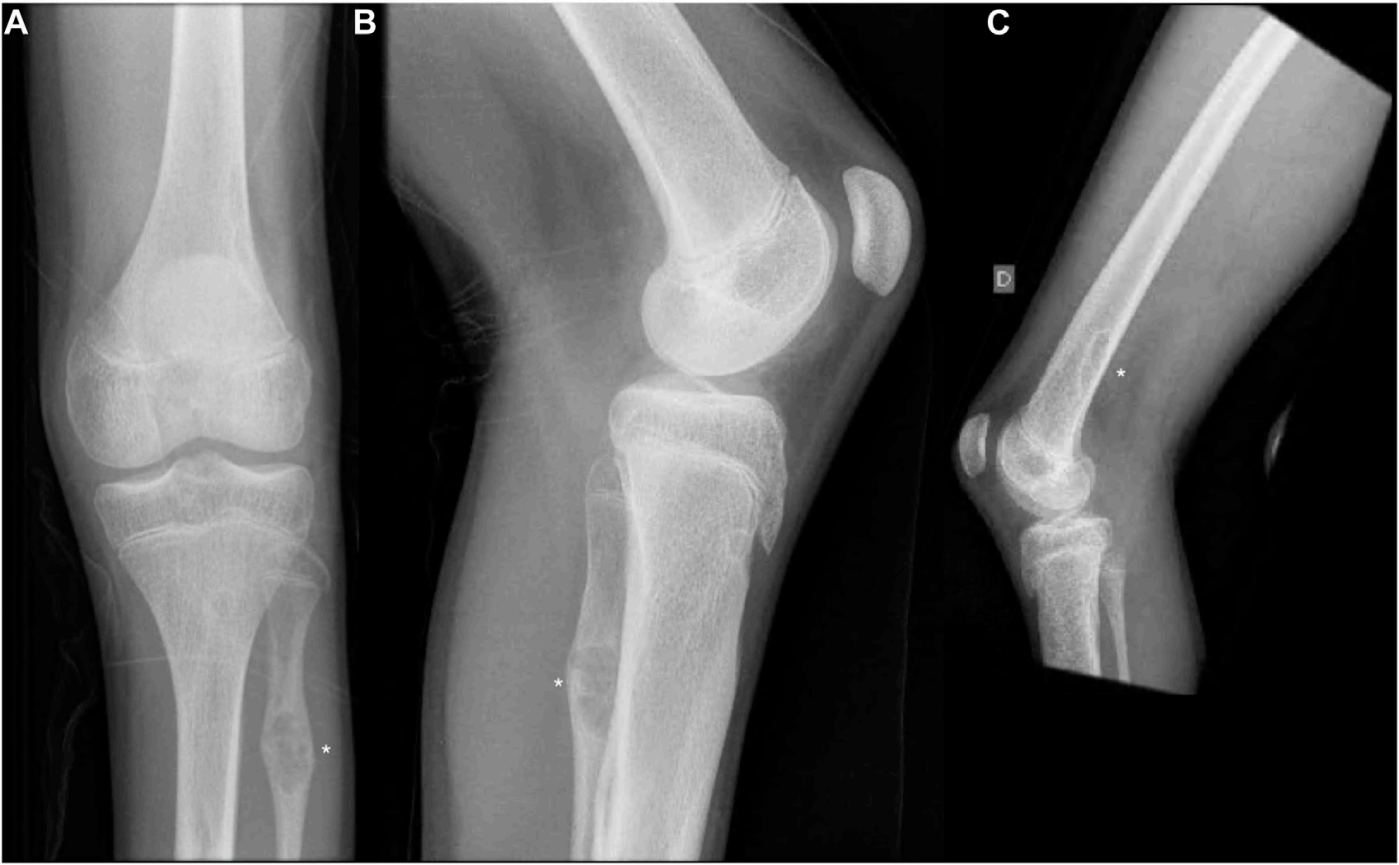
**(A,B)** Anteroposterior and lateral radiographs of the left knee showing non-ossifying fibroma in the distal femur and proximal fibula. **(C)** Lateral radiographs of the right knee showing non-ossifying fibroma in the distal femur. Non-ossifying fibromas are indicated by asterisks.

At the age of 14, he fell from a standing height and was diagnosed with a fracture of the distal diaphysis of the radius and ulna of the left upper limb. The fractures were treated conservatively. X-ray showed osteolytic changes in the distal part of the left radius (Figure 1). After 18 months, he suffered a second fracture in the same area. This time, X-ray imaging of the entire skeleton was performed, which, in addition to osteolytic changes in the area of the left radius, also showed osteolytic changes in the proximal part of the left fibula with fibrous changes after previously unnoted fracture and osteolytic changes in the distal part of the right and left femur (Figure 2). At the age of 16, he underwent MRI of the left lower extremity, which showed a benign lesion on the proximal fibula with a non-aggressive appearance, defined as a non-ossifying fibroma (NOF) or fibrous dysplasia. A biopsy was not performed. Laboratory tests showed the serum levels of calcium, inorganic phosphate, alkaline phosphatase, free T4 and T3, TSH, and PTH, were all normal. Bone mineral density was in the lower range of normal values.

At the last examination, at the age of 16 years, his height was 178.5 cm (0.25 z), weight was 43 kg (−4.37 z), head circumference was 58 cm (1.14 z). His physical exam demonstrated dolichocephaly, long face, strabismus, prominent nose, retrognathia, long, thin fingers with partial skin syndactyly of the second, third and fourth fingers and proximal interphalangeal flexion contractions, long toes, planus valgus, and thoracic scoliosis.

He had a moderate intellectual disability. He had regular medical check-ups at cardiology, nephrology, hematology and orthopaedic clinics.

The ASD and VSD spontaneously closed. He had Ebstein’s anomaly with moderate tricuspid valve regurgitation and left ventricular noncompaction cardiomyopathy with a normal-sized left ventricle with slightly impaired function.

He had bilateral renal dysplasia, with the right kidney being markedly more affected than the left. At the age of 6 years, the right kidney was already virtually non-functional and contributed about 5% of the total function. There was pyeloureteral stenosis on the right side and grade 2 vesicoureteral reflux, while the reflux on the left side had resolved spontaneously. Abdominal ultrasound examination at the age of 16 years revealed that the right kidney was smaller, measuring about 9 cm, with a uniformly thinned parenchyma measuring about 7 mm. The left kidney measured about 10, 5 cm, with reasonably wide parenchyma (about 15 mm).

He was followed up at the haematology clinic for thrombocytopenia, which was classified as mild [Platelets = 104 10^9/L (150–410^9/L)] at the last follow-up at the age of 16 years. The complete blood count with differential did not reveal any additional abnormalities.

He unexpectedly died during sleep at the age of 17.

Trio Exome sequencing (ES) was performed on genomic DNA of the patient and his parents using the Illumina Nextera Rapid Capture Expanded exome kit for exome enrichment. Sequencing was performed on the Illumina NextSeq 550 sequencer and the data analysed as previously described (Bergant et al., 2018). A median coverage of 211x was attained across exonic regions and 99.9% of exonic regions were covered with a minimum 20x coverage. Briefly, the data was analysed using a pipeline based on bwa alignment and variant calling in accordance with GATK best practice guidelines. The functional consequences of the resulting variants were predicted using the snpEff v.5.1 software and variants were annotated with bcftools 1.9 using the frequency data from the gnomAD exomes project v.2.1.1 and dbNSFP v3.0 resource (DePristo et al., 2011). We identified a novel *de novo* hemizygous splice-site variant NM_001145408.2: c.348+2_ 348+15del in *NONO* (PS2_MOD). The genome reference nomenclature of the variant was NC_000023.10:g.70511824_70511837del. The variant was absent from databases (gnomAD and the in-house population database) (PM2_SUP), was predicted to alter or cause deletion of the canonical sequence of the donor site adjacent to exon 5, possibly leading to the loss of functional protein product [NC_000023.10(NM_001145408.2):r.spl, NC_000023.10(NP_001138880.1):p.?, PVS1] and was classified as a pathogenic variant in accordance with the ACMG criteria (PVS1, PS2_MOD, PM2_SUP, Richards et al. (2015). In the classification process, the strength of PVS1 evidence was rated as very strong based on the published recommendations (Abou Tayoun et al., 2018), as the variant impacts a canonical splice site adjacent to a non-symmetric exon 5. Furthermore, the PS2 was weighted as moderate evidence in support of pathogenicity based on the ClinGen’s recommendations, due to a consistent albeit non-specific clinical presentation in the patient (ClinGen SVI recommendations for *De novo* criteria, version 1.1, available from https://clinicalgenome.org/working-groups/sequence-variant-interpretation/). Furthermore, the weight of PM2 was downgraded to PM2_SUP in line with the recent recommendations of the ClinGen SVI working group (Recommendation for Absence/Rarity Criterion PM2, version 1.0, available from https://clinicalgenome.org/working-groups/sequence-variant-interpretation/).

## 3 Discussion

Twenty-five patients with pathogenic variants of the NONO gene have been reported so far, of which 7 were prenatal cases (Mircsof et al., 2015; Reinstein et al., 2016; Scott et al., 2017; Carlston et al., 2019; Sun et al., 2020a; Sun et al., 2020b; Sewani et al., 2020; Coetzer and Moosa, 2022; Safi et al., 2022; Roessler et al., 2023). While in the prenatal period, the main clinical features are congenital heart defects and/or cardiomyopathy (7/7; 100%), the core phenotype in the postnatal period is developmental delay/intellectual disability (13/13; 100%), macrocephaly, corpus callosum anomalies (10/11; 91%), facial dysmorphic features, congenital heart defect (10/11; 91%) and left ventricular non-compaction cardiomyopathy (8/11; 73%). The patient presented here had all of these core phenotype clinical features. In addition, he had congenital thrombocytopenia, previously reported in three patient and renal abnormalities, previously described in a fetus that miscarried at 16 weeks and was found at autopsy to have renal agenesis Sewani et al., 2020. Although previously rarely reported, both congenital thrombocytopenia and renal anomalies are likely to be part of the clinical picture of patients with NONO-XLID and should be checked for.

Non-ossifying fibromas (NOFs) have not been previously reported in patients with NONO-XLID. The presented patient suffered recurrent fractures due to multiple NOFs at atypical locations. NOFs are benign tumours that typically appear in adolescence and then resolve. In less than 5%, NOFs are multifocal. The principal locations of NOF are metaphysis of the distal femur, proximal and distal tibia, but can also be found in the proximal humerus, fibula and distal radius (Mankin et al., 2009; Sakamoto et al., 2017). Pathologic fractures may occur with non-physiological loading or low-impact injuries and have been reported to occur in up to 20% of NOFs (Friedrich et al., 2021; Emori et al., 2022).

Multiple NOFs, with atypical location and fracture propensity, have been previously described in patients with neurofibromatosis type 1, and multifocal NOF have also been reported in patients with Jaffe-Campanacci syndrome and Oculoectodermal Syndrome (Peacock et al., 2015; Vannelli et al., 2020; Friedrich et al., 2021). All these disorders are known as RASopathies and are caused by germline or post-zygotic mosaic mutations in genes encoding RAS/MAPK signaling pathways components. Recently, NOF has been defined as a genetically driven benign neoplasm, caused by activation of Ras-MAPK signaling by somatic mutation in KRAS, FGFR1 and NF1 genes (Baumhoer et al., 2019), which also provides a causal genetic explanation for the occurrence of NOFs in RASopathies.

NONO-XLID is caused by hemizygeous pathogenic loss of function variants in the *NONO* gene and functional studies in patients showed loss of the NONO protein in patients’ cells (Mircsof et al., 2015; Roessler et al., 2023). NONO protein plays an important role in human tumorigenesis as either oncogene or tumour suppressor and is involved in many biological processes including cell proliferation, apoptosis, migration, and DNA damage repair (Feng et al., 2020). It has been suggested to play an important role in esophageal squamous cell carcinoma tumorigenesis by activation of the Erk1/2/MAPK pathway (Cheng et al., 2018) and is overexpressed in most cancers, while also lower NONO levels promote tumorigenesis in certain cancers (Pavao et al., 2001).

In summary, we report a patient with a diagnosis of NONO-XLID who suffered recurrent fractures due to multiple non-ossifying fibromas. The occurrence of multiple NOFs in unusual locations and recurrent fracturing, which is very rare in the general population, and the association of the *NONO* gene with tumourigenesis support the possibility that NOFs could be part of the clinical picture of NONO-XLID. In any case, the description of NOFs in a larger number of patients with NONO-XLID will be needed to confirm this. We suggest that patients with a *NONO* pathogenic variant who suffer low-force fractures should undergo skeletal X-ray imaging and a targeted search for lytic areas in the skeleton.

## Data Availability

The datasets presented in this study can be found in online repositories. The names of the repository/repositories and accession number(s) can be found below: https://www.ncbi.nlm.nih.gov/clinvar/variation/2429456/, submission name: SUB12837649; submission number: SCV003799180.

## Abbreviations

ASD, atrial septal defect; NOF, non-ossifying fibroma; PTH, parathyroid hormone; RAS/MAPK pathway, RAS/Mitogen Activated Protein Kinase pathway; T3, triiodothyronine; T4, thyroxine; TSH, thyroid stimulating hormone; VSD, ventricular septal defect; XLID, X-linked intellectual developmental disorder.
